# Enhanced Physiological Stress Response in Patients with Normal Tension Glaucoma during Hypoxia

**DOI:** 10.1155/2021/5826361

**Published:** 2021-04-07

**Authors:** Line Marie Dalgaard, Jeppe Vibæk, Rupali Vohra, Lars Thorbjørn Jensen, Barbara Cvenkel, Niels H. Secher, Niels Vidiendal Olsen, Miriam Kolko

**Affiliations:** ^1^Department of Drug Design and Pharmacology, University of Copenhagen, Copenhagen, Denmark; ^2^Department of Neuroanaesthesia, The Neuroscience Centre, Copenhagen University Hospital, Rigshospitalet-Glostrup, Copenhagen, Denmark; ^3^Department of Veterinary and Animal Sciences, University of Copenhagen, Copenhagen, Denmark; ^4^Department of Clinical Physiology and Nuclear Medicine, University Hospital of Herlev, Herlev, Denmark; ^5^Department of Ophthalmology, University Medical Centre Ljubljana, Ljubljana, Slovenia; ^6^Department of Anaesthesia, Copenhagen University Hospital, Rigshospitalet-Glostrup, Copenhagen, Denmark; ^7^Department of Biomedical Science, University of Copenhagen, Copenhagen, Denmark; ^8^Department of Ophthalmology, Copenhagen University Hospital, Rigshospitalet-Glostrup, Copenhagen, Denmark

## Abstract

**Purpose:**

To investigate whether patients with normal tension glaucoma (NTG) show an enhanced stress response to reduced oxygen supply compared to age-matched healthy controls, measured by serum adrenaline and endothelin-1 (ET-1) levels and changes in distal finger temperature.

**Methods:**

A thorough clinical characterization of patients with NTG and age-matched controls was performed prior to inclusion in the study. Twelve patients with NTG and eleven healthy controls met the inclusion criteria and were enrolled in the study. All subjects underwent a two-day investigation. Participants were randomly exposed to either hypoxia or normoxia during the first visit. Hypoxia or normoxia was induced for two hours through a tightly fitting face mask. In addition, the peripheral circulation was assessed with a thermographic camera. Blood samples were obtained before, during, and after hypoxia or normoxia to evaluate systemic stress molecules such as catecholamines and ET-1 levels.

**Results:**

In patients with NTG, reduced oxygen supply induced an increase in peripheral blood adrenaline (*p* < 0.05) and a decrease during recovery (*p* < 0.01). A difference in distal finger temperature was shown in patients with NTG under hypoxia compared to normoxia (exposure: *p* < 0.05; recovery: *p* < 0.05). Hypoxia induced an increase in peripheral blood ET-1 levels in both groups (NTG: *p* < 0.01; controls: *p* < 0.05).

**Conclusion:**

Patients with NTG had an enhanced physiological stress response as a consequence of hypoxia compared with age-matched controls. Although more studies are needed, the present study supports the involvement of vascular risk factors in the pathophysiology of NTG.

## 1. Introduction

Glaucoma is a progressive optic neuropathy characterized by loss of retinal ganglion cells (RGC) and their axons. Along with the loss of RGC, the disease is accompanied by a gradual loss of the peripheral visual field [1–4]. Glaucoma is the most frequent cause of incurable blindness and is estimated to affect approximately 111.8 million people by 2040 [5]. Together with aging, increased intraocular pressure (IOP) is recognized as the most important risk factor. However, patients may have an IOP in the normal range and still develop glaucomatous progression (normal tension glaucoma, NTG) [6]. Therefore, although the only existing treatments for glaucoma are IOP-lowering strategies, glaucoma is a multifactorial disease with many different risk factors [7]. Thus, identifying and characterizing other instigators are essential.

An increasing number of studies have suggested that glaucoma is a systemic disease that manifests in the inner retina, resulting in loss of RGC. The reason for this particular RGC vulnerability is the fact that these cells are especially dependent on a constant oxygen sand energy supply [8–12].

Consistent with this hypothesis, an increasing number of studies have shown that there is an association between vascular dysfunction and NTG [8–10, 12–14]. Vascular dysfunction is therefore thought to be an important risk factor for onset and progression of NTG [8–12, 15, 16].

Vascular dysfunction is defined as a condition in which the actual blood flow does not meet the requirements of the tissue for oxygen supply [13, 17]. This can result in overperfusion or underperfusion, which is basically due to an imbalance in the relationship between molecular vasoconstrictors and vasodilators. Dysregulated vascular constriction or dilation of the retinal blood flow may inevitably cause periods of hypo- and hyperperfusion [18–20], further escalating to hypoxic events and oxidative stress. As a result, such a cascade of reactions may contribute to glaucomatous neurodegeneration [11, 12, 21–25]. The body stabilizes blood flow by a highly regulated release of vasoconstrictive and vasodilative molecules [8–11]. This tight regulation of the vessel dynamics is termed autoregulation [18] and maintains flow by altering vessel diameter in response to changes in perfusion pressure [8, 12, 13]. Multiple factors influence autoregulation, such as CO_2_ levels, temperature, low grade inflammatory molecules, catecholamines, and ATP production [8, 9, 13, 15, 26]. Many of these variables are influenced by hypoxia and have been explored in animal experimental models of glaucoma [27–29]. However, to our knowledge, the relationship between decreased oxygen supply and these molecular changes has not been studied in patients with glaucoma.

Thus, the present study aimed to provide novel insight into systemic effect and molecular changes in response to reduced oxygen supply in patients with glaucomatous neurodegeneration compared to controls. Since we assume that all patients with glaucoma have multiple risk factors and since we were particularly interested in studying IOP-independent factors, we examined glaucoma patients with IOP within the normal range, where IOP is hypothetically a less significant risk factor. We thus examined patients with NTG and compared their response to systemic hypoxia with age-matched control subjects. We have previously shown that hypoxia reduces oxygen saturation levels in healthy test subjects and in patients with NTG within six minutes when they breathe 10 % oxygen [30]. With this human experimental model, hypoxia is used as a universal oxygen stress model. In the present study, we aimed to investigate whether patients with NTG have an enhanced stress response compared to healthy controls when exposed to reduced oxygen availability, including measuring various vascular parameters such as serum levels of catecholamines and ET-1 as well as distal finger temperature.

## 2. Methods

**Figure 1:**
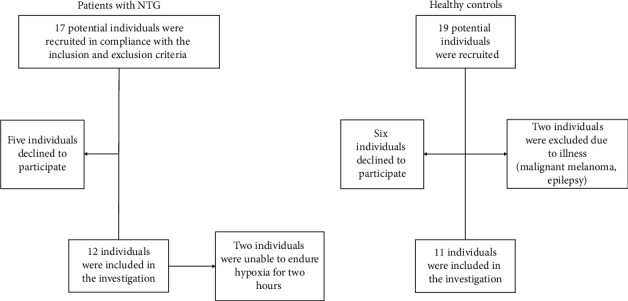
An overview of the inclusion of participants. A total of 12 patients with NTG were included, 10 of whom completed the study, in addition to 11 healthy controls. Excluded subjects did not meet the inclusion or did not want to participate.

A total of 23 eligible test subjects participated in the study between May 2015 and August 2016 [30]. The test subjects were assigned into two different groups: patients with NTG (12 participants) and age-matched healthy controls (11 participants) (Table 1 and Figure 1). Power and sample size calculations were based on an arterial vessel diameter from a previous study by Wong et al. [31]. In this study, the mean arterial vessel diameter was found to be 204.4 *μ*m with a standard deviation of 18.6. With a power of 80%, a *p* value of 0.05, and an allowed variation in diameter of 12%, a total of 9 individuals were required in each group. Other published works that required gas inhalation of volunteers have used similar sample size to that in this study [32]. All participants were ≥50 years old and nonsmokers. Patients with NTG were recruited by a glaucoma specialist through the Department of Ophthalmology at Zealand University Hospital, Roskilde. Control subjects were recruited via opticians to the study “Detecting Visual Fields Defects With Damato Multifixation Campimetry Online” [33] and were invited to participate in the present study.

This interventional case-control study was performed in compliance with the Declaration of Helsinki approved by the National Committee on Health Research Ethics (ethical protocol: H-2-2014-060). All participants received written information about the study and had the study verbally explained and provided both oral and written consent prior to participation. Inclusion criteria and exclusion criteria are summarized in Tables 2 and 3.

All subjects underwent two days of investigation. In random order, the visits included either normobaric hypoxia or normobaric normoxia. Hypoxia/normoxia was induced for two hours through a tight fitting face mask. The mask was connected via a Y-piece to a Douglas bag which was filled with either atmospheric air or a mixture of 10% oxygen and 90% nitrogen. We chose two hours of hypoxia induction to ensure a sustained effect on the cardiovascular system. This has been shown to be evident two hours after initial exposure [34]. Furthermore, two hours of hypoxia has previously been used to investigate endothelial function in healthy adults [35]. Previous studies have verified that the acute effect of hypoxia is abolished after 15 min, and we added a safety margin of further 15 min [36], resulting in a defined recovery period of 30 min after terminated hypoxia.

To ensure the safety of our participants, they were continuously monitored on both days of investigations with a three-lead ECG (M1166A model 66S, Hewlett Packard, Palo Alto, California, USA) and noninvasive blood-pressure measurement and pulse oximeter by a Nexfin monitor (BMEYE B.V., Amsterdam, Netherlands). We also encouraged our participants to let us know if they felt any discomfort. As an additional safety measure, the investigations were carried out at the Department of Anesthesiology, Rigshospitalet, Denmark, where we had anesthesiologists on call.

The two days of investigation were at least three weeks apart. All investigations were preceded by 12 hours of fasting.

### 2.1. Blood Samples

**Figure 2:**
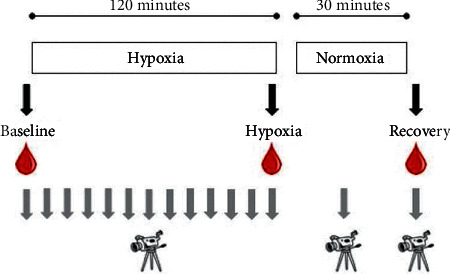
Blood samples were collected before, during, and after hypoxia. Thermographic images were obtained every tenth minute during hypoxia as well as 15 and 30 minutes after hypoxia.

Blood samples were collected from a peripheral vein on both days of investigation before, during, and after hypoxia/normoxia as shown in Figure 2. At each collection, 3 × 6 mL EDTA glasses were taken and put on ice. The glasses were centrifuged for 10 minutes at 4000 rpm, after which the supernatant was pipetted to Eppendorf tubes and frozen at −80°C until further analyses.

#### 2.1.1. Catecholamine Analysis

Catecholamine analyses were performed on serum from EDTA tubes at the Department of Clinical Biochemistry, Biolab, Rigshospitalet-Glostrup, Denmark, using a 2-CAT RIA kit according to the manufacturer's protocol (BA R-6500, Labor Diagnostika Nord GmbH & Co.KG, Nordhorn, Germany).

#### 2.1.2. Endothelin-1 Analysis

ET-1 levels were determined using an ET-1 ELISA kit (ab133030, Abcam, Cambridge, United Kingdom) following the manufacturer's protocol. Serum from EDTA tubes was collected from the −80°C freezer, thawed on ice, and extracted prior to analysis through Sep-Pak columns (Sep-Pak Vac C18 3cc, Waters Corporation, Milford, Massachusetts, USA) according to the manufacturer's protocol (ab133030, Abcam, Cambridge, United Kingdom). Nitrogen evaporation was used instead of a centrifugal concentrator under vacuum. The dried samples were stored at −20°C and reconstituted using 500 *μ*L of Assay buffer.

### 2.2. Thermographic Imaging

Thermographic imaging was carried out using the FLIR SC660 camera (FLIR Systems Inc., Wilsonville, Oregon, USA). Thermographic pictures of the dorsum of the left hand including all fingers were taken every tenth minute throughout the two hours of hypoxia/normoxia. In addition, a picture was taken 15 minutes and 30 minutes after the end of gas inhalation. The thermographic camera was set in a fixed position approximately one meter above the hand of the subject.

All investigations were performed in the same temperature-controlled room (22.5–23.5°C). In order to eliminate the influence of changing outside temperatures as well as possible Raynaud's phenomenon, both days of investigations were preceded by 30 minutes of preparations within the temperature-controlled room.

**Figure 3:**
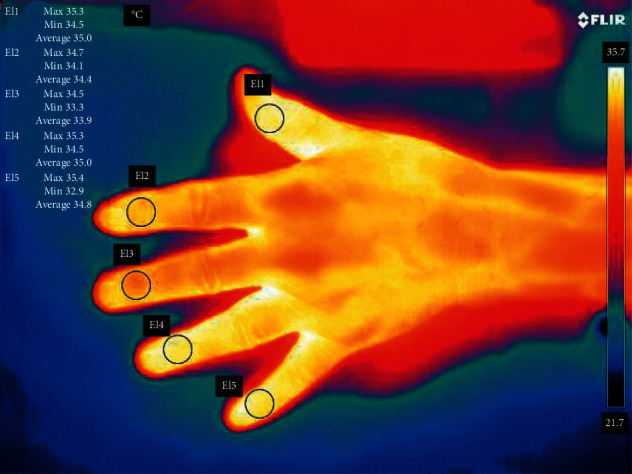
Thermographic image of a participant's hand. The circles represent the area in which the software calculates the mean temperature outlined in the left table. The circles were placed manually within the FLIR computer program. To the right, a scale of colors indicates that yellow and white symbolize warmer temperatures than blue and black.

The thermographic pictures were analyzed using the FLIR software in which a circle was added proximally to the cuticles of each finger (Figure 3) from which the average temperature was retrieved from the software. An average of the temperatures from the five fingers was set to the temperature of the hand at the according time.

### 2.3. Statistics

Statistical analyses were calculated using GraphPad software (GraphPad Prism version 7.0). Comparisons were analyzed by two-way ANOVA for comparing differences between baseline, hypoxia, and recovery among the two groups. One-way ANOVA with repeated measures taking pairing into account was used for comparing differences in time points within only one group (patients with NTG or age-matched healthy controls). In all analyses, *p* < 0.05 was considered statistically significant. Quantitative results are expressed as means ± SEM.

## 3. Results

### 3.1. Sustained Hypoxia in Patients with NTG and Healthy Controls

As we have previously reported, a significant decrease in pO_2_ and saturation during hypoxia was seen in both NTG patients and healthy controls leading to sustained hypoxia in both groups within 6 minutes of breathing 10% oxygen. There were no significant differences between the two groups at any time. We found no significant differences or changes in blood pressure either in the same group or between groups. There were no significant differences between the two groups at any time. We found no significant differences or changes in blood pressure either in the same group or between groups [30].

### 3.2. Hypoxia Induces an Increase in Adrenaline in Peripheral Blood in Patients with NTG

**Figure 4:**
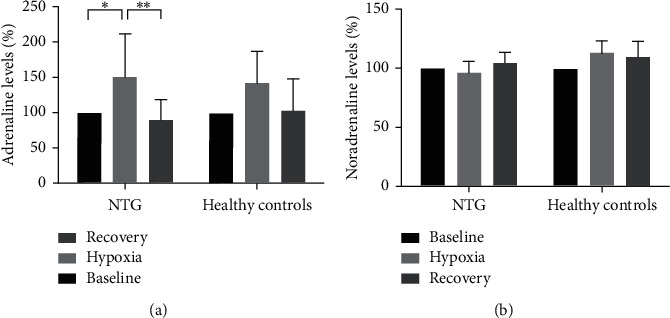
A relative increase in peripheral blood adrenaline was seen during hypoxia for patients with NTG. Patients with NTG also showed relative decrease in adrenaline during recovery. No significant changes were seen for age-matched healthy controls. No significant differences or changes were seen for noradrenaline (statistics: two-way ANOVA, Tukey's multiple comparisons test).

Peripheral blood adrenaline levels increased during hypoxia in both groups but only significantly in patients with NTG (to 151.3% ± 19.2 during hypoxia (*p* < 0.05) (Figure 4). Likewise, a decrease during recovery was seen in both groups, but the decrease was only significant in patients with NTG (to 89.3 % ± 9.5 during recovery, *p* < 0.01). There were no significant differences in peripheral adrenaline levels between the two groups during baseline, hypoxia, or recovery (Figure 4(a)). No significant changes or differences were seen for peripheral noradrenaline levels (Figure 4(b)).

### 3.3. Hypoxia Induces Elevated Temperature in the Distal Finger of Patients with NTG

**Figure 5:**
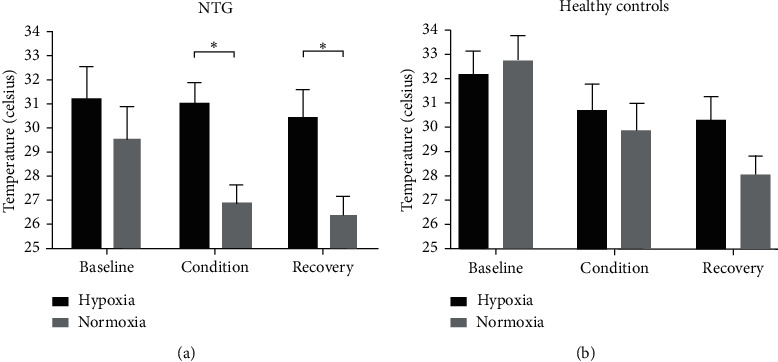
Overview of thermographic data. The distal finger temperature was significantly higher after two hours of hypoxia compared to two hours of normoxia, as well as during recovery, for patients with NTG. No significant differences were seen for age-matched healthy controls (statistics: two-way ANOVA, Sidak's multiple comparisons test).

When comparing the two days of investigation (hypoxia versus normoxia), patients with NTG showed higher distal finger temperature after two hours of gas inhalation with 10 % oxygen compared to atmospheric air (31.07°C ± 0.87 for hypoxia and 29.57°C ± 1.08 for normoxia, *p* < 0.05). This difference was also present during the recovery phase (30.46°C ± 1.16 for hypoxia and 26.40°C ± 0.77 for normoxia, *p* < 0.05) in patients with NTG (Figure 5(a)). However, no difference in distal finger temperature was seen in the age-matched control group when comparing the two conditions (Figure 5(b)). Patients with NTG tended to have lower finger temperatures compared with controls throughout both investigations; however, the decreased temperatures in patients with NTG were not significant.

### 3.4. Hypoxia Leads to Increased Serum Endothelin-1 Levels

**Figure 6:**
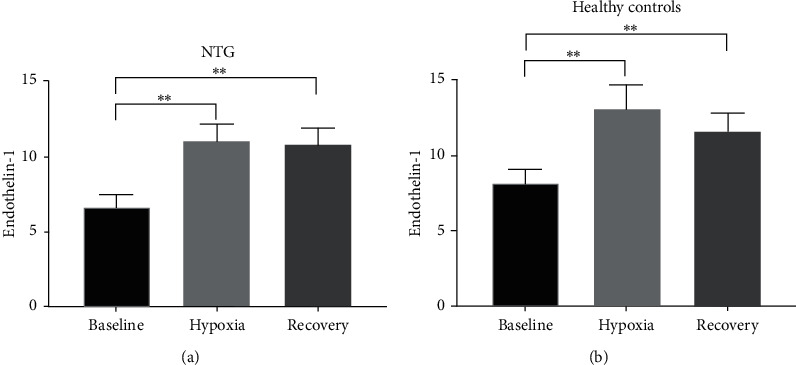
Overview of changes in serum levels of ET-1. ET-1 increased significantly during hypoxia in patients with NTG and in age-matched healthy controls. ET-1 levels remained high during recovery, creating a significant difference between baseline ET-1 levels and recovery in both groups (statistics: RM one-way ANOVA with Tukey's multiple comparisons test).

ET-1 levels increased during hypoxia in patients with NTG (from 6.68 mmol/L ± 0.82 at baseline to 11.05 mmol/L ± 1.14 during hypoxia, *p* < 0.01) (Figure 6(a)). Similarly, ET-1 levels also increased in healthy controls (from 8.23 mmol/L ± 0.87 to 13.27 mmol/L ± 1.61, *p* < 0.05) (Figure 6(b)). ET-1 levels only decreased slightly during recovery leading to a significant difference between baseline and recovery in both groups (NTG: 6.68 mmol/L ± 0.82 at baseline versus 10.81 mmol/L ± 1.15 during recovery, *p* < 0.01; controls: 8.23 mmol/L ± 0.87 at baseline versus 11.66 mmol/L ± 1.21 during recovery, *p* < 0.01). No significant differences were seen between the two groups.

## 4. Discussion

The vascular hypothesis, as a risk factor for glaucoma development and progression, suggests that RGC and their axons are prone to oxygen and nutrient insufficiency as a result of compromised blood flow. In support of this hypothesis, several studies have shown that hypoxia-related factors are upregulated in the eyes of glaucoma patients, indicating that levels of oxygen and nutritional supply fluctuate, which hypothetically will lead to oxidative stress and inflammation and ultimately glaucomatous neurodegeneration [11–13, 18–21, 27]. In this study, we have used a human experimental model to study how patients with glaucoma respond to systemic oxygen stress compared with age-matched test subjects. Although there is no significant difference in age between the two groups, the average age differs with 5 years between the two groups. The number of participants is small, and it is possible that this may have influenced the findings. With this model, we found that patients with NTG appear to have a higher systemic stress response in terms of greater changes in adrenaline levels during and after hypoxia compared with eye-healthy test subjects (Figure 4(a)) [8–12, 23, 24]. Furthermore, we found that temperature regulation is more fluctuating in patients with NTG compared to healthy controls. Our study suggests that patients with NTG are poorer at handling fluctuating oxygen availability, which may hypothetically result in increased levels of oxidative stress and inflammation which over time will lead to RGC neurodegeneration.

Previously, hypoxia has often been used as a metabolic stress model in *in vitro* studies of glaucoma [27–29], but, to the best of our knowledge, no previous studies have used an *in vivo* human model to study systemic changes that may be related to glaucomatous neurodegeneration. Whereas the present study verified increasing adrenaline in response to hypoxia in patients with NTG (Figure 4(a)), no significant increase was seen in the age-matched control subjects, implying that patients with NTG react more in response to metabolic stress compared to healthy control subjects.

Acute stress initiates a *fight-or-flight response* during harmful events, such as hypoxia, to promote survival. The response is facilitated through increase in adrenaline and manifests in increased blood flow and liberation of energy substrates for muscle action [37, 38]. In line with this, our recent study revealed a significant elevation in serum lactate from the patients with NTG [30], which has been attributed to being a prominent energy source in cells of the inner retina, including RGCs [39–41].

**Figure 7:**
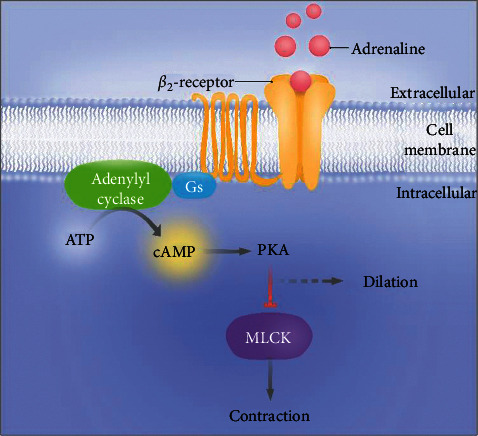
The *β*_2_ adrenergic receptor is a G-protein coupled receptor. Activation of the receptor by adrenaline leads to peripheral vasodilation through the Gs signal pathway. Activation of adenylyl cyclase leads to conversion of ATP to cAMP, which activates phosphor kinase A (PKA). Activation of PKA leads to deactivation of myosin light chain kinase (MLCK), which, when activated, leads to contraction. Thus, activation of PKA leads to vasodilation (©EyeTRU).

Adrenaline and noradrenaline are furthermore each known to stimulate both vasodilatory and vasoconstrictor responses in the same vascular bed, depending on their concentration and the distribution of adrenergic receptor subtypes in the vessel. There are two subtypes of receptors in blood vessels of clinical relevance: *α*_1_ elicits vasoconstriction and *β*_2_ induces vasodilation through the mechanism shown in Figure 7. Both adrenaline and noradrenaline have affinity for both receptors, but adrenaline has greater affinity for *β*_2_, while noradrenaline has greater affinity for *α*_1_. Although *α*_1_ is the predominant receptor subtype in the blood vessels [42], we found only a significant increase in adrenaline in response to hypoxia, suggesting a net activation of *β*_2_ receptors. Thus, our study suggests that hypoxia causes vasodilation, which may explain the increase in finger temperature in patients with NTG (Figure 5). Moreover, the increased finger temperature in patients with NTG indicates that patients with NTG are more prone to develop an acute stress response. In support of our study, Wierzbowska et al. have compared 24-hour ECG from NTG patients with healthy controls and found a higher ratio of low frequency to high frequency, indicating a shift towards sympathetic activity [43]. Furthermore, Flammer et al. demonstrated a link between primary vascular dysregulation syndrome (PVD) and NTG, highlighting lower hand temperature and an autonomic imbalance with sympathetic dominance as symptoms of PVD [15].

Despite the lack of autonomic innervation in the intraocular vascular system [44], it is likely that the systemic effects of adrenaline and noradrenaline play a role in the pathogenesis of glaucoma indirectly, if not directly [45–47]. In this context, Fitzgerald reports that systemic stress may lead to increased IOP [45]. In line with such systemic affection on retinal neurodegeneration, Horwitz et al. found that antiadrenergic antihypertensive drugs have a protective effect on the development of glaucoma [46, 47], which hypothetically may be explained by increased ocular perfusion. Thus, systemic levels of catecholamines may indirectly affect oxygen and energy supply to the inner retina and thereby play a role in the pathogenesis of glaucomatous neurodegeneration.

Since retinal vessels are autoregulated by ET-1 amongst others [8, 9, 13, 15, 26], we measured ET-1 in peripheral blood as a surrogate for the retinal ET-1 concentrations. ET-1 is a well-known vasoconstrictor in the eye and has repeatedly been demonstrated to play a key role in the regulation of ocular perfusion and hypothetically in the overall pathogenesis of inner retinal diseases [15, 48–51]. Circulating ET-1 can reach vessels in the optic nerve head (ONH) in two ways: (1) diffusion from the fenestrated choriocapillaris bypassing the blood-brain-barrier (BBB)/blood retinal barrier (BRB) or (2) through disrupted BBB/BRB. Disrupted BBB/BRB occurs both physiologically with aging and in response to neurodegeneration [15, 52, 53]. As a consequence of disrupted BBB/BRB, ET-1 can potentially freely access the vasculature supplying the optic nerve, and an increase in ET-1 in peripheral blood will lead to an increase in ocular ET-1 [15]. Our study showed a significant hypoxia-induced increase in serum ET-1 levels in both patients with NTG and controls (Figure 6). Previous studies have identified differences in ET-1 levels, when comparing patients with NTG to healthy controls. Li et al. have analyzed seven studies in a meta study, which showed higher plasma levels of ET-1 in the NTG group (mean difference of 0.6  pg/mL [*p* = 0.007, 95% CI = 0.17–1.04]) [14]. However, the present study was not able to replicate these findings, possibly due to deviations in recruitment criteria concerning clinical phenotyping, ethnicity of the test subjects, and so forth.

In summary, the present study introduces the concept of an enhanced hypoxia-induced stress response in patients with NTG which may be correlated to glaucomatous neurodegeneration. However, future studies are required to evaluate retinal vessel diameter in response to hypoxia and correlate findings to other blood stress markers to elaborate on vascular dysfunction and hypoxia-mediated stress responses in patients with glaucoma.

## Figures and Tables

**Table 1:** Characteristics of participants.

	NTG	Controls	*p* value
Age	70.3 ± SEM 1.5	65.6 ± SEM 2.3	0.09
Gender	Female: 6 (50%) Male: 6 (50%)	Female: 5 (45%) Male: 6 (55%)	0.83
BMI	23.6 ± SEM 1.0	25.8 ± SEM 0.8	0.11

**Table 2:** Inclusion criteria for patients with NTG.

Untreated intraocular pressure (IOP) never detected higher than 21 mmHg measured at different times of the day (8 AM–5 PM)
Open anterior chamber angles observed by gonioscopy
Glaucomatous cupping characterized by a violated ISNT rule (that normal eyes show a characteristic configuration for disc rim thickness of inferior ≥ superior ≥ nasal ≥ temporal)
Glaucomatous visual field loss by Humphrey perimetry or Octopus perimetry

**Table 3:** Exclusion criteria for patients with NTG and healthy age-matched controls.

Medical history including ocular trauma or eye conditions other than glaucoma involving the optic nerve
Significant systemic disease, e.g., hypertension, heart failure, hypercholesterolemia, diabetes mellitus, autoimmune diseases, and previous cerebral infract or bleeding
Individuals who were unable to cooperate during examination
Individuals below the age of 50 years
Individuals who smoke

## Data Availability

The blood sample and thermographic data used to support the findings of this study are available from the corresponding author upon request.
